# Usefulness of five-item and three-item Mental Health Inventories to screen for depressive symptoms in the general population of Japan

**DOI:** 10.1186/1477-7525-3-48

**Published:** 2005-08-08

**Authors:** Shin Yamazaki, Shunichi Fukuhara, Joseph Green

**Affiliations:** 1 Epidemiology and Exposure Assessment Section, National Institute for Environmental Studies, Tsukuba, Japan; 2 Department of Epidemiology and Healthcare Research, Graduate School of Medicine and Public Health, Kyoto University, Kyoto, Japan; 3 Office of International Academic Affairs, Graduate School of Medicine, University of Tokyo, Japan

## Abstract

**Background:**

The five-question Mental Health Inventory (MHI-5) is a brief questionnaire that can be used to screen for depressive symptoms. Removing the 2 anxiety-related items from the MHI-5 yields the MHI-3. We assessed the performance of the Japanese versions of the MHI-5 and MHI-3 in detecting depressive symptoms in the general population of Japan.

**Methods:**

From the population of Japan, 4500 people 16 years old or older were selected by stratified-random sampling. The Medical Outcomes Study 36-Item Short Form Health Survey (SF-36, which includes the MHI-5) and the Zung Self-rating Depression Scale (ZSDS) were included in a self-administered questionnaire. ZSDS scores of 48 and above were taken to indicate the presence of moderate or severe depressive symptoms, and scores of 56 and above were taken to indicate the presence of severe depressive symptoms. We computed the correlation coefficient between the ZSDS score and the scores on the MHI-5 and MHI-3. We also computed the sensitivity, specificity, and area under the receiver operating characteristic (ROC) curve.

**Results:**

Of the 3107 subjects (69% of the 4500 initially selected), 14.0% had moderate or severe depressive symptoms, and 2.0% had severe depressive symptoms as measured with the ZSDS. The correlations of ZSDS scores with MHI-5 scores and with MHI-3 scores were similar: -0.63 and -0.61, respectively. These correlation coefficients were almost the same whether or not the data were stratified by age and sex. For detecting severe depressive symptoms with the MHI-5, the area under the ROC curve was 0.942 (95%CI: 0.919 – 0.965); for the MHI-3, it was 0.933 (95%CI: 0.904 – 0.962).

**Conclusion:**

The MHI-5 and MHI-3 scores were correlated with the ZSDS score, and can be used to identify people with depressive symptoms in the general population of Japan.

## Background

Depression disorders are a major health problem in Japan. Depressive mood is associated with suicide in middle-aged workers [1], and the number of suicides has increased as economic conditions have worsened since 1998 [2]. Nonetheless, there are few studies of the prevalence of depression or of depressive symptoms in communities in Japan [3,4].

To assist in detecting depression or depressive symptoms, many screening questionnaires have been developed. Some of these have 20 to 30 items, take only a few minutes to complete, use the number of symptoms as the score, and have good performance to detect depressive state. Instruments that are even shorter but nonetheless have good performance to detect depressive state have also been developed [5-7]. One such questionnaire is the five-item version of the Mental Health Inventory (MHI-5) [6,7]. The MHI-5 is used as the "Mental Health" domain of the Medical Outcomes Study 36-Item Short Form Health Survey (SF-36). The SF-36 has been translated into Japanese [8], and the Japanese version has been validated for use in the general population of Japan [9], but the performance of the MHI-5 has not been evaluated in detail. In addition, two of the items in the MHI-5 are almost identical to two items in a scale developed to measure anxiety [10]. We hypothesized that removing those two anxiety-related items would result in a scale (the MHI-3) that performs as well as the MHI-5 in detecting symptoms of depression.

In this study, we compared the Japanese version of the MHI-5 and MHI-3 to the 20-item Zung Self-rating Depression Scale (ZSDS) [11], and assessed the performance of the Japanese versions of the MHI-5 and MHI-3 in detecting depressive symptoms among the general population.

## Methods

### Setting and participants

We used data that had been collected previously for a study of the validity of the Japanese version of the SF-36, and calculated national norm scores of all subscales of the SF-36 [8,9]. Details of the nationwide survey have been described previously [9]. Briefly, a total of 4500 people 16 years old or older were selected from the entire population of Japan by stratified-random sampling in 1995. A self-administered questionnaire was mailed, and the subjects were visited to collect the questionnaires. The SF-36, the ZSDS [11] (described below), and questions about demographic characteristics were included in the questionnaire.

The ZSDS consists of 10 positively worded items and 10 negatively worded items asking about symptoms of depression. Several studies have established the ZSDS as a reliable and valid instrument for measuring depressive symptoms [12-14]. The ZSDS scores were used to define four categories of the severity of depression: within normal range or no significant psychopathology (below 40 points); presence of minimal to mild depression (40–47 points); moderate to marked depression (48–55 points); presence of severe to extreme depression (56 points and above). These score ranges result from the studies of Zung [15] and Barrett et al [16]. The ZSDS has been translated into Japanese and studies of the validity of the Japanese version have been published [17]. Because the ZSDS is not a clinical diagnostic tool, subjects with high scores are said to have depressive symptoms rather than "depression."

Like the rest of the SF-36, the MHI-5 was administered as a paper-and-pencil questionnaire. The instrument contains the following questions: 'How much of the time during the last month have you: (i) been a very nervous person?; (ii) felt downhearted and blue?; (iii) felt calm and peaceful?; (iv) felt so down in the dumps that nothing could cheer you up?; and (v) been a happy person?' For each question the subjects were asked to choose one of the following responses: all of the time (1 point), most of the time (2 points), a good bit of the time (3 points), some of the time (4 points), a little of the time (5 points), or none of the time (6 points). Because items (iii) and (v) ask about positive feelings, their scoring was reversed. The score for the MHI-5 was computed by summing the scores of each question item and then transforming the raw scores to a 0–100-point scale [18].

Items (i) and (iii) are almost identical to 2 items in the Zung Self-rating Anxiety Scale [10]. To make a scale that is even shorter than the MHI-5 and is focused on depression we removed those two anxiety-related items. Thus, the MHI-3 comprised only (ii), (iv), and (v) above. Possible scores on the MHI-3 ranged from 3 to 18 points.

### Statistical methods

First, we computed the correlation coefficient (Pearson's) between the ZSDS scores and the scores on the MHI-5 and the MHI-3. We computed the sensitivity, specificity, and area under the receiver operating characteristic (ROC) curve. Analysis of ROC curves has been described in detail and ROC analysis is used extensively in health-related diagnostics [19,20]. ROC analysis can be used to study the performance of diagnostic or screening tests across a wide range of sensitivities and specificities. For example, it can be used to compute the sensitivity (the true-positive rate) and specificity (the true-negative rate) for any specified test score. The area under the ROC curve (AUC) is an index of the amount of information the test provides over its entire scoring range [21,22]. In general, an AUC can range from 0.5, which indicates a test with no information, to 1.0, which indicates a perfect test. The "gold standard" criteria for diagnosing depression are considered to be those of the Diagnostic and Statistical Manual of Mental Disorders (DSM) [7]. In this study, because we could not interview all subjects, we used, instead, scores on the ZSDS. For each of the three categories of the severity of depressive states (ZSDS scores of 40 or higher), we computed the AUC of each of the five items, the MHI-5, and the MHI-3. To define the cut-off points, we first considered each of the actually measured MHI-5 scores as a possible cut-off point. For each score, we took the sum of the sensitivity and the specificity. The score with the highest sum was used as the cut-off point. One cut-off point was determined for each of the three levels of severity defined by ZSDS scores (mild, moderate, and severe).

## Results

The nationwide survey targeted 4500 people, and 3395 (male: 1704; female: 1691) responded to the questionnaire (75% response rate). Of these 3395 individuals, 3107 (male: 1573; female: 1534) completed all of the items on the ZSDS. The mean score on the MHI-5 was 72.8 (SD = 19.1). The mean scores on the MHI-5 for respondents of different demographic categories are shown in Table 1. These mean scores ranged from 68.5 to 76.6. Almost 23% of the respondents had ZSDS scores indicating mild depressive symptoms, 12% had scores indicating moderate depressive symptoms, and 2% had scores indicating severe depressive symptoms.

**Table 1:** MHI-5 scores by demographic categories

	N (%)	Score of the MHI-5
	3107 (100)	Mean (SD)
Sex		
Male	1573 (51)	73.31 (18.63)
Female	1534 (49)	72.32 (19.55)
Age (years)		
<30	619 (20)	70.17 (18.47)
30 – 39	506 (16)	72.50 (17.47)
40 – 49	665 (21)	72.38 (20.28)
50 – 59	617 (20)	74.22 (18.60)
60 – 69	479 (15)	75.21 (19.11)
≥70	221 (7)	73.23 (21.09)
Annual household income (million yen)		
<3	385 (12)	69.37 (20.83)
3 – 4.9	670 (22)	71.87 (19.08)
5 – 6.9	685 (22)	72.62 (19.38)
7 – 9.9	648 (21)	73.72 (18.27)
10 – 11.9	228 (7)	74.57 (18.78)
≥12	266 (9)	76.63 (16.72)
Missing values	225 (7)	73.30 (19.4)
Schooling		
Junior high school	613 (20)	72.64 (19.88)
High school	1426 (46)	72.97 (18.88)
Junior college, college, or higher	1028 (33)	72.84 (18.85)
Missing values	40 (1)	69.95 (20.88)
Marital status		
Single	622 (20)	70.04 (18.89)
Married	2227 (72)	73.74 (18.8)
Separated	28 (1)	75.43 (16.86)
Divorced	65 (2)	68.66 (20.91)
Widowed	152 (5)	72.18 (22.27)
Missing values	13 (0)	70.00 (19.71)
Occupational status		
Full time worker	1610 (52)	73.05 (18.25)
Part time worker	299 (10)	74.27 (17.96)
Retired	164 (5)	72.51 (22.74)
Unemployed	171 (6)	69.34 (21.95)
Homemaker	533 (17)	73.06 (19.71)
Student	226 (7)	73.36 (18.77)
Other	83 (3)	68.48 (21.49)
Missing values	21 (1)	70.86 (16.64)

The correlations of ZSDS scores with MHI-5 scores and with MHI-3 scores were similar: -0.63 and -0.61, respectively. These correlation coefficients were almost the same whether or not the data were stratified by age and sex (Table 2).

**Table 2:** Correlations of ZSDS scores with MHI-5 and MHI-3 scores, by demographic category

	MHI-5	MHI-3
All	-0.634	-0.614
Sex		
Male	-0.634	-0.610
Female	-0.635	-0.618
Age (years)		
<30	-0.653	-0.643
30 – 39	-0.686	-0.685
40 – 49	-0.619	-0.591
50 – 59	-0.576	-0.549
60 – 69	-0.635	-0.608
≥70	-0.698	-0.671
Annual household income (million yen)		
<3	-0.666	-0.638
3 – 4.9	-0.612	-0.596
5 – 6.9	-0.642	-0.642
7 – 9.9	-0.637	-0.602
10 – 11.9	-0.654	-0.642
≥12	-0.562	-0.554
Missing values	-0.613	-0.551
Schooling		
Junior high school	-0.612	-0.579
High school	-0.637	-0.617
Junior college, college, or higher	-0.651	-0.636
Missing values	.	.
Marital status		
Single	-0.661	-0.638
Married	-0.624	-0.602
Separated	.	.
Divorced	.	.
Widowed	-0.658	-0.642
Missing values	.	.
Occupational status		
Full time worker	-0.618	-0.601
Part time worker	-0.533	-0.509
Retired	-0.741	-0.711
Unemployed	-0.714	-0.692
Homemaker	-0.646	-0.636
Student	-0.680	-0.646
Other	.	.
Missing values	.	.

**Figure 1:**
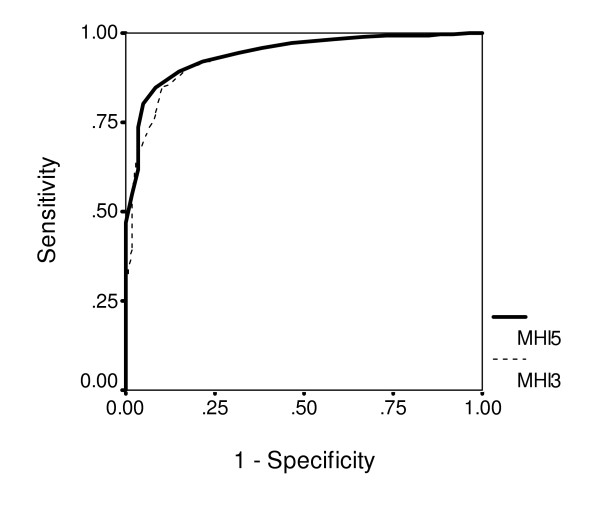
ROC curves of the MHI-5 and MHI-3 for detecting severe depressive symptoms (ZSDS above 55).

With ZSDS scores as the basis for classifying depressive symptoms, ROC analysis allowed us to evaluate the performance of the MHI-5 and the MHI-3. The AUC values are shown in Table 3, and other performance characteristics are shown in Table 4. We also evaluated the performance of each of the MHI-5 question items individually (Table 3). For the individual items, the range of "cut-off scores" was determined by the range of each question's response options: from "none of the time" to "all of the time." The best-performing item for detecting severe depressive symptoms was the one asking about the frequency of "feeling downhearted and blue". That item had a sensitivity of 0.88 and a specificity of 0.77 (based on a score of 4 points or less). The AUC of the MHI-3 was only slightly lower than that of the MHI-5 (Figure 1).

**Table 3:** ROC analysis of individual MHI-5 items, the whole MHI-5, and the MHI-3, by severity of depressive symptoms

Items and scales	Severity of depressive symptom (range of ZSDS scores)					
	Mild, moderate, or severe (40 through 80)		Either moderate or severe (48 through 80)		Severe (56 through 80)	
	AUC	(95% CI)	AUC	(95% CI)	AUC	(95% CI)
(i) Nervous person	0.696	(0.677–0.716)	0.707	(0.680–0.734)	0.826	(0.774–0.879)
(ii) Down in the dumps	0.713	(0.694–0.733)	0.741	(0.714–0.769)	0.862	(0.813–0.910)
(iii) Calm and peaceful	0.745	(0.726–0.764)	0.755	(0.728–0.782)	0.845	(0.797–0.892)
(iv) Downhearted and blue	0.739	(0.720–0.758)	0.748	(0.721–0.776)	0.898	(0.855–0.941)
(v) Happy person	0.747	(0.729–0.765)	0.738	(0.711–0.765)	0.858	(0.811–0.905)
						
MHI-5*	0.810	(0.793–0.826)	0.819	(0.795–0.843)	0.942	(0.919–0.965)
MHI-3†	0.800	(0.783–0.817)	0.803	(0.779–0.828)	0.933	(0.904–0.962)

Values shown are areas under the ROC curves (AUC), and their 95% CIs, for three levels of depressive symptoms as measured by ZSDS scores. *The MHI-5 includes all 5 items. †The MHI-3 includes only items ii, iv, and v.

**Table 4:** Performance of the MHI-5 and MHI-3 for detecting depressive symptoms

	Mild, moderate, or severe depressive symptoms (ZSDS scores of 40 or higher)		Moderate or severe depressive symptoms (ZSDS scores of 48 or higher)		Severe depressive symptoms (ZSDS scores of 56 or higher)	
Prevalence	37%		14%		2%	

Instrument	MHI-5	MHI-3	MHI-5	MHI-3	MHI-5	MHI-3

(cut-off score)	(68)	(14)	(60)	(13)	(52)	(11)
						
Sensitivity	71.5%	76.4%	74.7%	77.1%	91.8%	90.0%
Specificity	79.1%	71.1%	80.0%	71.8%	84.6%	84.2%
Positive predictive value	66.7%	60.8%	37.1%	30.8%	10.8%	10.4%
						
Negative predictive value	82.5%	83.7%	95.1%	95.1%	99.8%	99.8%

Using the MHI-5, the prevalence of severe depressive symptoms (cut-off: 52 points) was 17%, that of moderate or severe depressive symptoms (cut-off: 60 points) was 28%, and that of mild, moderate, or severe depressive symptoms (cut-off: 68 points) was 40%.

## Discussion

These data show that the MHI-5 and MHI-3 scores were each correlated with the ZSDS score and had good screening accordance with the ZSDS in the general population of Japan. We also found that the MHI-3 performs almost as well as the MHI-5. The best-performing single item was the one asking about "feeling downhearted and blue," which was also the case in the US [6]. The usefulness of the MHI-5 is consistent with results of a study done in the US [6]. Each scale and each item performed best as a detector of severe depressive symptoms, but each also contributed some information even for detecting moderate and mild depressive symptoms (Table 3). Both scales performed better than did any item alone.

Because prevalence affects positive predictive value, the latter was lowest for severe depressive symptoms and was highest for mild, moderate, and severe depressive symptoms (Table 4). For all levels of symptom severity, the positive predictive values of the MHI-3 were similar to those of the MHI-5, and for severe depressive symptoms they were nearly identical (10.8% and 10.4%) (Table 4).

A previous study showed that the prevalence of mood disorders (major depression, bipolar disorders, and dysthymia) as measured using the DSM criteria in Japanese people 20 years old and older was 3.1% [4]. On the other hand, 37% of the sample in the present study had mild, moderate, or severe depressive symptoms as measured using the ZSDS. People in whom depression is diagnosed using the DSM criteria are probably only a small number of those who report at least some depressive symptoms. In a previous study that also used the ZSDS, the prevalence of mild depressive symptoms among Japanese male workers was 45% [23], which is similar to that in our study.

In addition to its performance as shown in the present ROC analysis, an advantage of the MHI-5 may be the fact that it is part of the SF-36. The reason is that the possibility of a Hawthorne-type effect (i.e. an effect on study participants that results from their knowing that they are being studied) can be an obstacle to screening for depressive state. Specifically, the subjects' responses on a mental-health screening instrument may be affected by their knowledge that they are subjects in a study of mental health. Embedding the mental-health screening instrument in a more general survey, as the MHI-5 is embedded in the SF-36, could help minimize any such effect.

While the results of this study may be useful for public-health purposes, surveys done in primary-care settings could provide information that is more directly applicable to clinical work. Also, it should be kept in mind that ZSDS scores alone cannot be used to diagnose clinical depression. Studies using psychiatrist-diagnosed depression in addition to ZSDS scores would provide further information about the utility of the Japanese version of the MHI-5.

Another limitation is that the data set was obtained from a 1995 survey. Further studies are needed to confirm the performance of the MHI-5 and MHI-3 using data obtained in recent years.

In conclusion, the MHI-5 and MHI-3 scores were correlated with the ZSDS score, and can be used to identify people with depressive symptoms in the general population of Japan.

## List of abbreviations

AUC: area under the ROC curve; MHI-5: the five-item version of the Mental Health Inventory; MHI-3: those 3 of the MHI-5 questions that were thought to be most directly related to depression; ROC: receiver operating characteristic; SF-36: the Medical Outcomes Study 36-Item Short Form Health Survey; ZSDS: the Zung Self-rating Depression Scale.

## Authors' contributions

SY: analysis of the data, interpretation of results, manuscript writing; SF: initiation and study design, supervision, collection of data; JG: supervision, interpretation of results, manuscript writing.
